# Advocacy organizations and nutrition policy in Nigeria: identifying metrics for enhanced efficacy

**DOI:** 10.1093/heapol/czac037

**Published:** 2022-04-28

**Authors:** Danielle Resnick, Kola Anigo, Olufolakemi Mercy Anjorin

**Affiliations:** Brookings Institution, Global Economy and Development Program, 1775 Massachusetts Avenue NW, Washington, DC 20036, USA; Ahmadu Bello University, Food and Nutrition Unit, Department of Biochemistry, Samaru Campus, Community Market, Zaria 810211, Nigeria; University of Ibadan, Department of Human Nutrition, Ward XI, Nw8, Ibadan, Nigeria

**Keywords:** Advocacy, food fortification, metrics, Nigeria, nutrition, public policy

## Abstract

Advocacy organizations have played a significant role in the field of nutrition in recent years. However, why are some advocates viewed as more effective than others? This paper derives metrics for assessing advocacy efficacy by first drawing on key insights from the nutrition and public policy scholarship. A set of metrics is proposed to capture the constitutive elements of three concepts that often emerge as critical from that literature: organizational capacity, strong networks and external outreach. Based on a survey of 66 nutrition stakeholders in Nigeria, including at the federal level and within the states of Kaduna and Kano, the metrics are then applied to a set of advocacy organizations within the country. We show that the metrics can provide insights into why some advocacy organizations are perceived as more effective than others by policymakers. Specifically, we find that geographical reach, the share of budget allocated to advocacy, action plans with clear objectives, large networks that include government and non-governmental policy champions, multiple media and dissemination outputs and numerous training events collectively increase nutrition advocates’ visibility to, and influence on, policymakers. Although the metrics are subject to further testing in other country settings and need to be interpreted based on a country’s underlying policy system, they offer a useful starting point for more systematic, comparative advocacy analysis and learning within the nutrition field and beyond.

Key messages

- Advocacy has helped increase public attention to nutrition policy in recent years.
- Through a case study focused on nutrition advocacy organizations at both the federal and state levels in Nigeria, this paper identifies why some advocates are more effective than others.
- Based on 66 interviews with a broad range of stakeholders, metrics are derived to operationalize three common components deemed essential to advocacy efficacy: organizational capacity, strong networks and external outreach.
- The metrics allow for a more systemic approach for comparing advocacy efficacy in both the nutrition field and other public policy domains.

## Introduction

Over the last decade, nutrition has received elevated attention in international and national policy arenas. For instance, nutrition’s importance is affirmed by inclusion in the Sustainable Development Goals and elevated by financial commitments from global leaders through initiatives such as Nutrition for Growth. Policy advocacy has played a significant role in this shift by raising awareness about underlying causes of malnutrition, emphasizing associated economic and social costs and creating targets by which governments should be held accountable (Pelletier *et al.*, 2013; Mejía Acosta and Haddad, 2014; te Lintelo *et al.*, 2016). However, in the crowded arena of nutrition advocacy, why are some advocates viewed as more effective by policymakers than others?

To address this question, this paper reviews existing literature on the components of effective nutrition advocacy and proposes a way of operationalizing these components. Subsequently, it discusses how the proposed metrics fared in the case of Nigeria. After describing how data was collected from 66 key informants, including advocacy organizations, government officials, donors and academics, we show how policymakers perceive the efficacy of different advocacy organizations. Variation in perceived effectiveness of different organizations is then assessed vis-à-vis the proposed metrics to identify which are most robust and should be considered by those interested in leveraging advocacy in the nutrition arena.

## Theoretical context

There are numerous definitions of advocacy (see te Lintelo *et al.*, 2016). For instance, Gen and Wright (2013, p. 165) note that advocacy consists of ‘intentional activities initiated by the public to affect the policymaking process.’ Pelletier *et al.* (2013, p. 86) define advocacy as ‘a continuous and adaptive process of gathering, organizing, and formulating information into argument, to be communicated to decision-makers through various interpersonal and media channels, with a view to influencing their decision…’ Cullerton *et al*. (2018, p. 83) noted that advocacy is ‘The process of undertaking active interventions with the explicit goal of influencing government policy.’ At their core, these and other definitions all see advocacy being an ongoing and interactive process, targeted primarily at policymakers.

Many different stakeholders can be advocates for particular positions, including multilateral and bilateral donors and the business community. However, we define advocacy organizations as non-profit agencies whose core mandate involves promoting particular causes, ideas and norms (Keck and Sikkink, 1998) or who participate in such promotional activities in addition to providing direct services, including technical training, community education and program implementation (see Kimberlin, 2010). Advocates may have achievements in multiple ways, such as by building trust within local communities or fostering dialogue on polarizing issues. However, following Raynor *et al*. (2009), this paper defines advocacy efficacy as attaining some type of policy outcome, such as through identifying a policy problem, shifting the policy agenda, changing policy design, or facilitating policy implementation. Such an explicit focus on policy outcomes corresponds with much of the empirical scholarship on nutrition advocacy discussed below.

While there has been an expansion of advocacy evaluation methods over the last decade (e.g. Gen and Wright, 2013), these approaches aim to guide advocacy organizations to self-reflect on their goals, benchmarks and strategies. However, there is less attention to how advocacy organizations can be assessed in relation to one another. Therefore, we elaborate on three interrelated components that extant literature collectively and consistently suggests are necessary—although by no means sufficient depending on the enabling environment—for effective advocacy: organizational capacity, strong networks and external outreach.

### Organizational capacity

Advocates in any field require a minimum set of human and fiscal resources to build momentum and scale-up activities (McCarthy and Zald, 2002). Comparative case studies of nutrition policy and broader advocacy show that organizations’ achievements rely on, *inter-alia*, staff with relevant skills and knowledge about the specific policy domain who can determine which research and information is credible (Elbers and Kamstra, 2020; Pelletier *et al.*, 2013). If the aim is to influence policy at the subnational level as well, the organization requires the presence of trained staff outside the capital city (Harris *et al.*, 2016). Others find that advocates are likely to be more successful if they have strategic capacity, including a clear, tactical vision, demonstrated by time-bound objectives that are supported by action plans or an organizational strategy (Reisman *et al.*, 2007). As Prakash and Gugerty (2010) observe, advocacy organizations are motivated not only by normative concerns, but also by instrumental ones related to their organizational survival and growth. Therefore, financial resources are another key sub-element of organizational capacity, which need to be available for the duration of the advocacy effort, whether a one-time campaign or a more long-term engagement (McCarthy and Zald, 2002; Shiffman, 2016).

### Strong networks

Individual organizations can enhance their resilience and adaptability by establishing strong coalitions or networks, which have proved essential for advancing policy change in spheres as diverse as nutrition, education and the environment (e.g. Di Gregorio *et al.*, 2019; Harris, 2019; Sabatier and Weible, 2007). One of the major public policy perspectives is the Advocacy Coalition Framework (ACF) advanced by Sabatier and Jenkins-Smith (1993). In this view, such coalitions are united by a set of policy beliefs within their particular policy subsystem, such as nutrition policy and a geographical focus, such as a state or country. The ACF argues that coalitions are founded on a hierarchy of beliefs. Specifically, deep core beliefs are overarching normative assumptions about how governments should act. Policy core beliefs refer to the expected role of actors in addressing the deep core beliefs while secondary beliefs are narrow and focused on policy mechanisms (Sabatier and Weible, 2007). For the nutrition community, there is unity in deep core beliefs that tackling malnutrition is a fundamental responsibility of governments and increasingly of the private sector (Development Initiatives, 2020). There is also shared policy core beliefs that malnutrition needs to be tackled through multi-sectoral interventions. However, disagreements occur at the secondary belief level regarding the policy instruments, such as behavior change campaigns, ready-to-use therapeutic food (RUTF), or fortification strategies, that should be prioritized.

A critical consideration is the composition of the network. A dense network ensures the incorporation of diverse skills and policy contacts, potentially leading to more creative policy solutions and a more holistic understanding of the enabling environment. However, the trade-off is that more diversity can also contribute to conflicting perspectives among network members (Shiffman, 2016; te Lintelo *et al.*, 2016). Disputes can result in mixed messaging to decisionmakers, leading to either policy inertia or contradictory policy interventions. A formal governance structure can assist with inter-organizational cooperation by mitigating potential conflicts among network members and enhancing collective action among members (Shiffman, 2016).

Such networks often require building alliances with a range of policymakers to better tailor policy options to the decisionmaking environment (Cullerton *et al.*, 2016). Often, building linkages with a powerful policy champion has helped advocates establish local legitimacy and build momentum (Balarajan and Reich, 2016; Resnick *et al.*, 2018). Policy champions often are ‘insiders’ in the reigning power structures and derive their influence from their status and power. Such champions should not only be limited to elected officials but also include senior bureaucrats from relevant ministries (Pelletier *et al.*, 2013; te Lintelo *et al.*, 2016) and public figures with popular legitimacy among the broader population (McCarthy and Zald, 2002).

### External outreach

To both build networks and exert policy influence, advocates need to pursue a variety of external outreach activities that communicate their positions to targeted stakeholders. Such communications must be derived from evidence that is viewed as credible by the local communities in which they are operating (Harris *et al.*, 2017). Moreover, the way in which advocates frame their positions and their policy issue is essential. Frames ideally reorient thinking about an issue by identifying a problem and a solution as well as suggesting who is affected and the consequences of inaction (Chong and Druckman, 2007).

The selected frame needs to not only resonate with external actors, including political elites (Gillespie *et al.*, 2013; Shiffman *et al.*, 2016), but also reflect a consensus within the broader advocacy network about the policy beliefs discussed above. Depending on the issue, such framing strategies can be difficult for nutrition communities to agree on, as shown by Pelletier *et al*. (2012) in Guatemala and Harris (2019) in Zambia. Shelley (2012) argues that framing over obesity has led to a ‘policy cacophony,’ creating confusion among decisionmakers about which interventions should be prioritized. Frames that work best appear to convey simple messages digestible to both the public and politicians and build on values respected in the target society (Freudenberg *et al.*, 2009; Cullerton *et al.*, 2016).

Advocates also need to disseminate their work and positions in multiple ways to engage in frame ‘amplification’ (Cullerton *et al.*, 2018). This requires both inside and outside tactics (Dellmuth and Tallberg, 2017); the former involves direct interaction with decision makers through phone calls, email exchanges and closed-door meetings while the latter focuses on mobilizing public opinion through awareness campaigns, op-eds and other modalities to indirectly pressure decisionmakers. For instance, the Advocacy Working Group in Uganda pursued its efforts around stunting policy through policy briefs, newspaper articles, a documentary on local television stations and radio call-in programs (Pelletier *et al.*, 2013). Active forms of policy engagement, such as high-level events, parliamentary briefings or journalist training workshops, can help to reinforce a particular frame and communicate evidence about complex nutrition issues (Harris *et al.*, 2017).

### Summary

**Figure 1.**
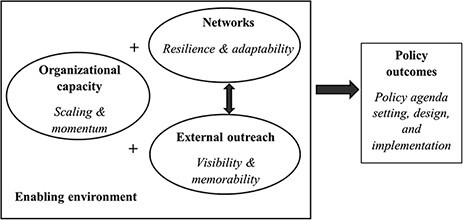
Pathways of Advocacy Efficacy

Figure 1 summarizes how these factors contribute to policy outcomes and interact with each other: organizational capacity allows for scaling and sustained momentum, strong networks provide resilience and adaptation to new policy developments and outreach ensures visibility and memorability in an increasingly crowded marketplace of ideas and priorities. Each of these factors is embedded in and influenced by the enabling environment, inclusive of the external institutional, political and legal settings in which advocates operate (e.g. Harris *et al.*, 2017; Resnick *et al.*, 2021). Yet, given that many dimensions of the enabling environment are constant in our case study due to the use of a single country case study, the current paper only focuses on metrics for advocacy that are more internal to organizations and over which they can exert agency to directly change. Similarly, while there are other potential factors that shape advocacy efficacy, such as seizing windows of policy opportunity and honing skills as a policy entrepreneur (Raynor *et al.*, 2009; Cullerton *et al.*, 2018), such dynamics can be idiosyncratic and difficult to measure at the organizational level.

## Materials and methods

The preceding section shows there has been significant learning across diverse contexts about what makes advocacy effective in general and particularly with respect to nutrition policy. However, there is less effort to operationalize these lessons, which prevents advocates and their supporters from determining where they should invest resources to improve their efficacy. Moreover, there is often a lack of information about whether some of these dimensions of advocacy are more salient and consequential than others.

To address this gap, we analyzed the nutrition advocacy landscape in Nigeria where 37% of children under five are stunted (National Population Commission NPC and ICF, 2019), exceeding the African average of 30% (Development Initiatives, 2020). In addition, 68% of Nigerian children between 6 and 59 months are anemic and only 29% of infants under 6 months of age are exclusively breastfed (National Population Commission (NPC) and ICF, 2019). The importance of nutrition is widely acknowledged by different government ministries, and there are at least 19 nutrition-relevant national policies within the country (Vanderkooy *et al.*, 2019). The advocacy community is also extremely rich; one study found that there were more than 200 international and domestic non-profit and non-governmental organizations working in the nutrition domain (CS-SUNN and UNICEF, 2017).

Key informant interviews (KIIs) were conducted with 66 stakeholders to identify which advocacy organizations were most effective at influencing policymakers and, in turn, whether those organizations had specific characteristics that correspond to the three sets of factors reviewed above, i.e. organizational capacity, strong networks and external outreach. Since Nigeria is a federal country, many policy interventions are formulated at the national level but implemented at the state and local government area (LGA) levels. Thus, explicit attention was given to both levels, with a focus on the states of Kaduna and Kano. These two states in Nigeria’s northwest region are where many advocacy organizations are concentrated because they fare much worse on malnutrition indicators than states in the south of the country. For instance, child stunting prevalence ranges from 46–56% in Kaduna state and as high as 57–66% in Kano. Comparative ranges in states such as Delta, Lagos or Cross River are 14–24% (National Population Commission (NPC) and ICF, 2019).

The KIIs were conducted according to a detailed and consistent interview guide that is publicly available upon request. The identified stakeholders were selected from a list provided by a longstanding West Africa nutrition initiative led by one of the author’s institutes. We then shortlisted those that were still operational and located in Kaduna, Kano and/or the Federal Capital Territory of Abuja and focused on those with a focus on either infant and young child feeding (IYCF) or food fortification advocacy. These two areas are not only key priorities in Nigeria’s National Policy on Food and Nutrition but also involve appealing to different groups of policymakers. As such, advocacy efficacy can be assessed vis-à-vis stakeholders within the broad nutrition community as well as by those who work in one of the two specific nutrition domains. This purposive sampling ensured that our conclusions did not just reflect the views of one sub-community within the nutrition advocacy landscape.

In addition to conducting interviews in the capital of each state, health and agricultural departments in two LGAs each (Giwa and Kachia in Kaduna, Bichi and Wudil in Kano) were selected in consultation with each state’s nutrition officer and to reflect geographical variation within each state (i.e. an LGA from the North and South of each state). LGA departments are frontline nutrition service providers and those selected were already implementing Community Infant & Young Child Feeding and Community Management of Acute Malnutrition interventions, ensuring that LGA stakeholders would be knowledgeable enough to speak about health advocacy and government support for IYCF and food fortification. This process was important for ensuring that our findings did not simply reflect the perspectives of a narrow group of actors, i.e. national policymakers in the capital city, but rather accounted for the opinions of a broader range of actors in the policy process.

The interviews occurred from October to December 2019, and as shown in Table 1, spanned five main categories. While other studies have looked at stakeholder advocacy in Nigeria (e.g. Allcock and Barker, 2012), this study differed by approaching respondents bilaterally rather than in a workshop setting. By conducting face-to-face interviews, respondents could be more forthcoming about which advocacy organizations they felt were more effective. Appendix Table A1 provides a full list of stakeholder organizations that were interviewed, and Appendix Table A2 offers a full elaboration of organizational acronyms.

**Table 1.** Distribution of interviewees

	**Number**	**Share (%)**
Stakeholder group		
Advocacy organization	23	34.33
Government	28	42.4
Donor	4	6.1
Media	3	4.6
Research/consultant community	8	12.1
Total	66	100
Geographical distribution		
Federal	26	39.4
Kano	22	33.3
Kaduna	18	27.3
Total	66	100

## Results

### Perceived efficacy of advocacy organizations

**Figure 2.**
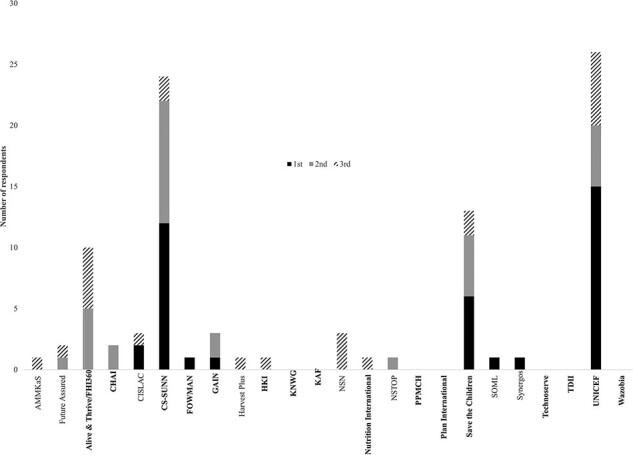
What are the top three nutrition advocacy organizations that you perceive as most effective in Nigeria/this state?

Our main outcome variable is perceived effectiveness of advocacy organizations by other, non-advocacy organizations, including donors, government decision makers and implementers and private sector actors. This was assessed in two ways. First, these non-advocacy respondents were requested to name the top three most effective nutrition advocacy organizations in either Nigeria or their state. Figure 2 illustrates the range of organizations that were identified. Those organizations in boldface were those that were also included in the survey as respondents while those in non-boldface were not. The figure highlights that organizations such as UNICEF, Save the Children and FHI 360 were perceived as the most effective, in addition to the Civil Society Scaling-Up Nutrition in Nigeria (CS-SUNN) non-profit, which is a hybrid organization that includes international and domestic nutrition advocacy organizations.

**Figure 3.**
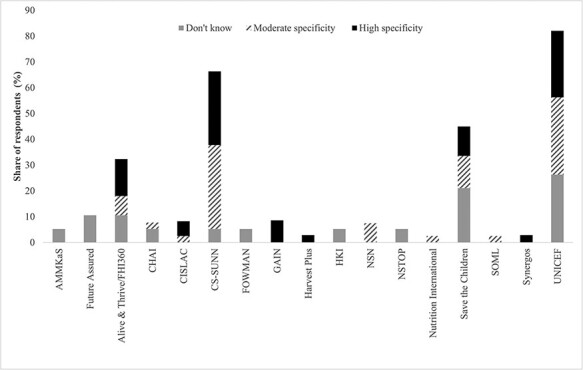
Share of respondents who can identify advocacy organization accomplishments in area of nutrition policy formulation, by level of specificity

Second, the same set of respondents were asked whether they could attribute a policy accomplishment to the advocacy organizations that they viewed as most effective. Figure 3 illustrates responses per organization based on a three-point coding of the interviewees’ qualitative answers. For instance, if a respondent could not attribute any accomplishment to an organization they designated as effective, that was coded as ‘don’t know.’ Answers that were relatively generic, such as ‘Domestication of food and nutrition policy in the state’ or ‘Generate discussion on policy review,’ were coded as providing ‘moderate specificity.’ Detailed responses, including ‘Part of the team that ensured six months of maternity leave’ or ‘brought forth the issue of hidden hunger which is now a serious policy objective of the Federal Ministry of Health,’ are coded as ‘high specificity.’ Generally, the organizations viewed as more effective in Figure 2 also have a larger share of respondents who can identify accomplishments with high specificity in Figure 3. In other words, respondents did not simply gravitate towards certain organizations because of name recognition, but they could associate specific actions with those organizations. Although we did not carry out detailed case studies to confirm each organization’s objective achievements, we believe that this approach captures how well advocates are making their positions and interventions known to a diverse range of policy actors.

Combined, Figures 2 and 3 illustrate that while some organizations are very visible and perceived as highly effective, others lie at the other end of the spectrum. To understand why, we follow best practice for comparative case study research (George and Bennett, 2005; Seawright and Gerring, 2008) and focus on six organizations that fall at differing levels of perceived effectiveness. Specifically, we are following the ‘diverse case’ study method for hypothesis testing, which Seawright and Gerring (2008) define as selecting a minimum of two cases that represent extreme values on the dependent variable as well as additional cases that represent the mean or median. Because this method encompasses the full range of variation, it is likely to enhance the representativeness of the sample cases to others in the broader population of cases.

At the one extreme, UNICEF and CS-SUNN were identified as among the most effective organizations in the country on nutrition. For instance, one observer noted that ‘The totality of the nutrition policies that we have had in Nigeria was led by UNICEF. The first policy, the second and the third was led by UNICEF, including the establishment of the coordination structures’^1^ while CS-SUNN.

‘Played a very critical role in the development of the nutrition policy and the multisectoral food and nutrition plan of action…They created an opportunity for us to know the funding gaps at the state level.’^2^ At the other extreme, no one identified the KAF Care Foundation or the Partnership for the Promotion of Maternal and Child Health (PPMCH), even by stakeholders in Kano state where those organizations are based. FHI360 and Nutrition International (NI) provide interim cases between these two extremes.

To understand the variation across these six cases, the next section applies the concepts reviewed earlier—organizational capacity, strong networks and external outreach—and shows which metrics could be used to reflect those concepts in a meaningful way. Table 2 above summarizes the metrics in general terms while Tables 3 through 5 provide details for each of the six organizations as well as the interview questions used to elicit responses. To enhance transparency and avoid assumptions of an omitted variable bias, we provide the range of data collected but boldface those metrics that demonstrate the greatest salience in explaining variance in perceived efficacy.

**Table 2.** Summary of concepts, sub-components, and metrics for assessing advocacy efficacy

Concept	Sub-component	Metric
Organizational Capacity	Human resource capacity	Number of staff in country
		**Number of staff with relevant technical skills**
		**Geographical reach of activities**
	Financial capacity	**Budget estimated *ex-ante* for specific advocacy activities**
		**Share of budgeted activities covered by existing funding**
	Strategic capacity	Longevity of organization
		Existence of an action plan or strategy
		**Action plan has clear objectives**
		Objectives are time-delimited
Networks	Composition of network	**Number of organizations in network**
	Formal governance structure	Number and type of modalities for inter-organizational cooperation (e.g. MoU, monthly meetings, etc.)
	Policy champion(s)	**Does the network have a government champion?**
		**Does the network have a non-government champion (e.g. celebrities, sports figures, politicians’ spouses)?**
	Cohesion	Number of points of agreement and disagreement in the network
External outreach	Credible evidence	Data/research that shapes advocacy position is peer-reviewed, authoritative and/or publicly available
	Common frame	An overarching frame can be identified that drives the advocacy position
	Dissemination in multiple formats	**Number of different media efforts in a year (e.g. newspaper op-eds, radio appearances, documentaries, social media)**
		**Number of different materials produced (e.g. policy briefs, reports)**
	Policy engagement	**Number of training events with journalists, parliamentarians, bureaucrats, and civil society organizations**

Notes: Boldface indicates greater explanatory power in explaining variation in efficacy across organizations.

**Table 3.** Organizational capacity metrics and comparisons

Sub-component	Metric	Interview Question	CS-SUNN	FHI360	KAF	NI	PPMCH	UNICEF
Human resource capacity	Number of staff in country	How many individuals work in this organization?	Between 10-20	Between 20-50	Between 20-50	Less than 10	Between 10-20	Between 20-50
	**Number of staff with relevant technical skills**	How many staff in this organization have experience and training in nutrition?	25	34	10	3	20	33
	**Geographical reach of activities**	Including this one, how many offices do you have in Nigeria?	24	5	4	1	1	10
		In approximately how many LGAs in Nigeria do you have activities?	44	28	7	0	1	95
Financial capacity	**Budget estimated *ex-ante* for specific advocacy activities**	Approximately how much of the budget, as a percentage, goes towards advocacy activities rather than for salaries or overhead costs?	65%	Unknown	55%	20%	60%	75%
	**Share of budgeted activities covered by existing funding**	Of the activities that are planned in an average year, approximately what percent can be covered by existing resources rather than anticipated appropriations?	80%	100%	30%	85%	Unknown	40%
Strategic capacity	Longevity of organization	How many years has this organization been in existence in Nigeria?	Between 5-7 years	Between 3-5	More than 7	More than 7	Between 5-7 years	More than 7
	Existence of an action plan or strategy	Does your organization have a document that outlines the accomplishments it wants to achieve?	Yes	Yes	Yes	Yes	Yes	Yes
	**Action plan has clear objectives**	If so, are there specific objectives outlined in the document? If yes, please provide an example these objectives	Mobilize non-state actors to strengthen coordination of nutrition and ensure capacity to implement nutrition in Nigeria.	Increase the rate of exclusive breast-feeding in targeted states.	Implement program on IYCF	Avert stunting among children 0 to 23 months.	Improve maternal health	Increase access to services an information for children, adolescents, mothers, and women, particularly in vulnerable, deprived areas so they adopt appropriate nutritional practices to prevent and treat malnutrition.
	The objectives are time-delimited	Are the objectives intended to be achieved by a specific date or timeframe? Please provide an example	No	Yes, by 2020	Yes, to be achieved by 2019	Yes, to reach 23 million women of re-productive age, adolescent girls, and children under-five in Nigeria by 2024.	This is an annual goal	Yes, 5 year strategic document.

### Organizational capacity

Organizational capacity can be disaggregated into three sub-components: human resource capacity, financial capacity and strategic capacity. These issues were observed as essential for advocacy in Nigeria: ‘The question is do organizations have the time and budget to advocate in a way that will establish the minimum number of contacts points needed? This requires funding and human resources.’^3^

For human resource capacity, having a sufficient ratio of staff with experience in nutrition compared to the number of employees within the organization feasibly allows for greater outreach, as does the number of offices and programs For UNICEF, CS-SUNN and FHI 360, having offices in both the capital and in multiple states contributed to their higher recognition by both federal government stakeholders and their counterparts in Kaduna and Kano states (see Table 3). Not surprisingly, more offices are correlated with more activities at the LGA level as well.

The two financing metrics need to be examined in tandem; ideally, advocacy efforts could be more impactful if a higher share of resources can be allocated to this goal *and* if organizations already possess those resources rather than rely on anticipated appropriations. While NI claimed they had 85% of their needed resources in an average year, only 20% is intended for advocacy. By contrast, UNICEF targets three-quarters of their resources for advocacy, even if they report having less than half of needed funding already in place within an average year.

As seen in Table 3, the length of an organization’s existence does not play an obvious role in explaining its perceived efficacy, and all six organizations note that they have an organizational strategy. The main difference is the specificity of their goals. KAF only claims that it has a program on IYCF and PPMCH notes it supports maternal health but neither organization provides details on the way in which it is promoted. FHI 360, NI and UNICEF all have very specific outcomes they plan to promote in certain constituencies (e.g. women, children), and FHI 360 and UNICEF have targeted geographical areas of focus (e.g. certain states, vulnerable areas).

### Strong networks

To identify the composition of advocacy networks, all 23 advocacy organizations in the survey were asked to identify the names of up to three organizations with which they partner most frequently to advance their objectives (see Table 4). Imposing a ceiling reduces the likelihood of respondents listing large numbers of minor partners and concentrates attention to the most important.

**Table 4.** Network metrics and comparisons

Sub-component	Metric	Interview Question	CS-SUNN	FHI360	KAF	NI	PPMCH	UNICEF
Composition of network	**Number of organizations in network**	Can you please share with us the names of up to 3 organizations with which you partner most frequently to advance your organization’s objectives?	FHI360 UNICEF Save the Children	CS-SUNN UNICEF NGF	Vitamin Angels SFH	UNICEF UNFPA HKI	CISLAC CS-SUNN	FHI360 CS-SUNN BMGF
Formal governance structure	Number and type of modalities for inter-organizational cooperation (e.g. MoU, monthly meetings, etc.)	For each of the organizations mentioned above, do you have a formal governance arrangement that guides your engagement, such as a board or regularly scheduled meetings?	Yes Yes Yes	No Yes No	No Yes	Yes Yes Yes	Yes Yes	Yes Yes Yes
		For each of the organizations mentioned above, what are the main activities in which you jointly engage?	Maternity leave protection and videos to promote exclusive breastfeeding Trainings and meetings with government officials to promote uptake of micronutrient supplementation Contact government officials	Reviewed 5 years’ nutrition budget at the national level and in 5 states. Host public events Contact government official	Host public events Contact a government official	Contact a government official Contact a government official Contact a government official	Host public events Host public events	Host public events Discuss ways to promote adoption of the National Food and Nutrition Plan by the states. Also, advocate for domestic resource mobilization. Evidence generation for members of the Nutrition Donor Group.
Policy Champion(s)	**Does the network have a government policy champion?**	Which government ministry, department, or agency, at either the federal, state, or LGA level, do you perceive as the most receptive to your advocacy activities?	Federal Ministry of Budget and Planning and corresponding State level budget ministries	Ministry of Health at Federal level, Ministry of Women’s affairs at the state level	Nutrition department in the State Ministry of Health	National Primary Health Care Development Agency	Kano State Ministry of Planning and Budget	Kaduna State Planning and Budget Commission, Federal Ministry of Budget and National Planning
	**Are there additional non-governmental champions with which the network liaises?**	Are there other prominent public figures (politicians, celebrities, sports figures, etc.) that also engage with your network and support your activities? If so, please elaborate	Aisha Buhari, Governors Forum Secretariat, wives of governors of Kaduna and Niger	Wives of state governors, Senator Ibrahim Oloriegbe	No	Sanusi Lamido Sanusi, Aisha Buhari	Sanusi Lamido Sanusi	Sanusi Lamido Sanusi, Aisha Buhari, Hajia Ummi El-Ruffai
Network cohesion	Areas of agreement and disagreement in network	What are the main points of agreement related to nutrition policy in Nigeria within this network of advocacy organizations?	People agree that malnutrition is a problem, the causes of malnutrition, and the need for things to change.	Early initiation of breastfeeding, exclusive breastfeeding for 6 months as well as the implementation of Breast Milk Substitute code.	Don’t know	We agree on need for policy on routine vitamin A supple-mentation and that there are too many Maternal and Child Health Week (MNCHW) interventions	Improve the nutrition situation in Kano	The thousand (1000 days) days focus.
		What are the main points of disagreement related to nutrition policy in Nigeria within this network of advocacy organizations?	Focusing on treatment of severe acute malnutrition without the preventive component. There is a disagreement in the amount of money budgeted for RUTF as opposed to preventive activities.	We don’t provide RUTF or do CMAM, like other organizations.	None	MNCHW online training modules.	None	There is a divide on whether the focus should be on treatment versus prevention. Also, there is a debate on fortification of certain food vehicles.

**Figure 4.**
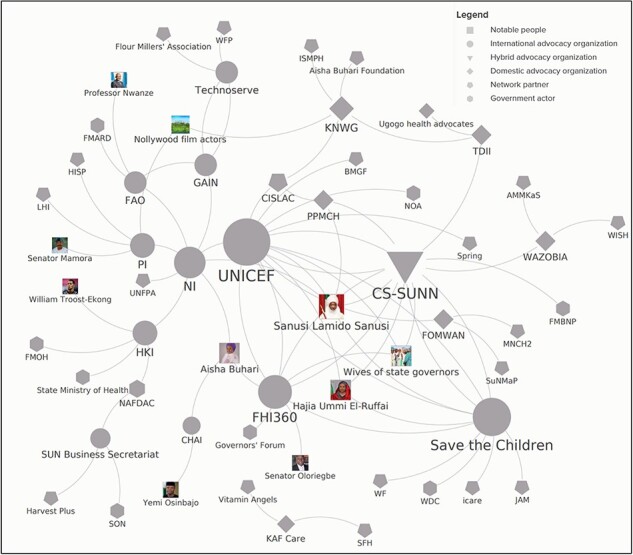
Network of Advocacy Organizations

Figure 4 maps the responses listed in Table 4 to provide a visual interpretation of the nutrition advocacy network. The size of the circles approximates the ‘degree centrality,’ which counts the number of links by each organization in a network. The shapes of the advocacy organizations indicate whether they are international (circle), domestic (diamond), or hybrid (triangle) entities. UNICEF is clearly the partner that most organizations mentioned as a partner, followed by CS-SUNN and FHI360. By contrast, PPMCH and KAF could only identify two advocacy organization partners, and KAF was not identified as a partner by any other organizations in the sample. Despite not having a high level of recognition by policy stakeholders, NI has a large number of advocacy partners, and spans the divide between the IYCF and food fortification communities. This reflects the organization’s transition in mandate in 2017: ‘[We] used to focus only on micronutrients. With the change of name to Nutrition International, the organization recently expanded to broader nutrition issues such as adolescent nutrition and institutional coordination.’^4^

However, the intensity of the network activities is not equivalent. For instance, both UNICEF and CS-SUNN work with their partners to engage in both inside tactics directly with the government and outside tactics with communities on specific issues of breastfeeding, budget allocations, resource mobilization and micronutrient supplementation.

As noted earlier, policy champions are central to networks’ legitimacy and visibility. We assessed policy champions from two perspectives. The first focused on institutional policy champions, particularly government ministries. To improve perceived effectiveness, more policy champions are better than fewer, and creating linkages at multiple levels in Nigeria theoretically increases the likelihood of influencing both policy formulation and implementation. CS-SUNN, FHI 360 and UNICEF have found ministerial partners at these dual levels. Yet, instead of noting a public health or nutrition champion, CS-SUNN and UNICEF view bureaucratic actors within budget and planning as major supporters for their activities. This is significant because when all 66 survey respondents were asked which government institution is most important for nutrition policy in Nigeria, 45% (30 respondents) identified either the Federal Ministry of Finance, Budget and National Planning (FMFBNP) or the State Planning and Budget Commission. A key reason for this is because the National (state) Committee on Food and Nutrition is housed within the Federal (state) budget ministries, which convenes the committees’ meetings, and has been critical for pushing for nutrition interventions to be costed within the federal and state budgets. Moreover, the federal MFBNP leads multi-sectoral coordination around the National Policy on Food and Nutrition in Nigeria and reports directly to the Vice-President on nutrition activities (World Bank, 2018). In other words, CS-SUNN and UNICEF partnered with a government actor with substantial power in the decision-making space in Nigeria, reflecting an understanding of the underlying policymaking system within the country and states. By contrast, KAF lacked any government champions in its network while PPMCH pointed to working with the National Orientation Agency (NOA) whose mandate encompasses issues beyond nutrition, including electoral violence and the environment.

Advocates also were asked about their linkages with specific individuals, including politicians, celebrities and other prominent public figures, who support their activities. Notably, KAF did not identify any such champions of this type. However, the other organizations work with the wives of state governors, particularly Kaduna state governor’s wife, Hajia Ummi El-Ruffai, as well as the Nigerian first lady (Aisha Buhari) and the former Emir of Kano and former Central Bank Governor (Sanusi Lamido Sanusi) (see Figure 4). These are three of the most active, well-known public figures in the nutrition sphere in Nigeria: El-Ruffai established in 2017 the Kaduna State Emergency Nutrition Action Plan (KADENAP) to fast track the work of ministries that deal with nutrition, women and children’s health, Sanusi frequently speaks about the scourge of malnutrition on the country’s development, and Buhari established the Future Assured Foundation, which focuses on child well-being.

As noted earlier, while density can be favorable for a network, the downside is that too many participants with different perspectives may affect cohesion. For KAF and PPMCH, no key areas of disagreement within their networks could be identified, likely reflecting the narrowness of their networks. PPMCH notes that its network agrees on the need to improve nutrition in Kano but provides no specificity for how members believe this should be achieved. This can be problematic for identifying areas of commonality that can be used as a springboard for action. By contrast, UNICEF’s network’s focus on the first 1000 days is specific enough to focus on the urgency of early childhood interventions, thereby serving as a unifying, policy core belief. This can help overcome divisions within its network over secondary beliefs, including whether to prioritize treatment (e.g. ready-to-use therapeutic foods) or prevention (e.g. IYCF) of malnutrition in certain circumstances. Both FHI360 and NI have narrower axes of agreement focused on policy levers they see as most effective to promote nutrition, including IYCF and vitamin A supplementation, respectively. The potential trade-off is that they can offer focused targets for government action at the expense of engaging cooperatively with a broader range of advocates.

### External outreach

As elaborated earlier, external outreach is most effective when it involves communicating messages based on credible evidence that are framed appropriately and delivered through multiple written, audiovisual media and in-person activities. The credibility of evidence can depend on whether there is a process for ensuring its rigor and transparency, including a process of peer review by experts or availability for public scrutiny. As shown in Table 5, FHI360, NI, CS-SUNN and UNICEF rely heavily on national survey data, such as publicly accessible Demographic Health Surveys and the National Nutrition and Health Survey. Three of these also conduct their own in-house data collection where needed. KAF conveyed a limited source of data outlets that inform its advocacy work.

When developing a frame for mobilization, actionable outcomes with clear processes are more useful because they suggest a clear policy position that decision makers can digest. While all six organizations provided a frame, the positions of KAF and PPMCH are quite vague and offer no actions. NI provides an aspirational frame but does not demonstrate how to reach the goal for which it strives. On the other hand, UNICEF identifies actions—domestic resource mobilization and policy/strategy development—but did not articulate the ultimate objective for such actions. Of the six organizations, FHI360 offers a clear objective and processes with measurable targets.

**Table 5.** External outreach metrics and comparisons

Sub-component	Metric	Interview Question	CS-SUNN	FHI360	KAF	NI	PPMCH	UNICEF
Credible evidence	Data/research that shapes advocacy position is peer-reviewed, authoritative, and/or publicly available	Where do you obtain data, research, and information to substantiate the policies that you advocate?	National Demographic and Health Survey (NDHS), National Nutrition and Health Survey (NNHS), and Multiple Indicator Cluster Surveys (MICS)	Facilities and communities where Alive and Thrive works. We also use data from NDHS and NNHS.	Kano Ministry of Health; UNICEF fact sheet	National Information System, NNHS, NDHS or administrative data from NPHCDA, global reports like the Lancet series. For IFA and Zinc project, the organization carried out its own research to generate data.	Health Management Information System, NDHS, Health facilities data	National survey data, routine data available from the line Ministries. UNICEF collects parallel data at global level like peer reviewed journals, WHO global database, UNICEF nutri-dash database.
Common frame	An overarching frame can be identified that drives the advocacy position	Could you identify an overarching narrative that drives the advocacy position? If so, please elaborate.	The need for urgent interventions to reduce the current level of malnutrition.	To increase the number of lives of children saved in Nigeria through infant and young child feeding practices	Reduce the rate of malnutrition in Nigeria	A world where everybody is free of hidden hunger, and ensuring that everybody particularly children and adolescent girls reaches their highest potential.	Reduce maternal malnutrition	Improve domestic resource mobilization, and policy and strategy development.
Dissemination in multiple formats	**Number of different media efforts in a year (e.g. newspaper op-eds, radio appearances, documentaries, social media)**	How many different media outreach activities do you pursue on average in a year?	Radio appearances, TV appearances, Media round tables at national level twice a year, events for international days like the World Food Day and World Breastfeeding Week, state-level media events, and press releases	Newspaper op-eds, radio, TV appearances, blogs	Newspaper op-eds	Radio appearances	Radio and TV appearances	Newspaper op-eds, radio, TV appearances, blogs, social media
		Do you have an operational website?	Yes	Yes	No	Yes	No	Yes
	**Number of different materials produced (e.g. policy briefs, reports)**	What are the different types of outputs that your organization produces, if any?	Quarterly newsletters, “Nutrition Tuesdays” on Twitter when people ask questions about nutrition and we respond.	Research reports (e.g. on National Maternity Entitlement), Advocacy Toolkits, Field data	Databases	Working papers	Policy and issue briefs, working papers	Reports, policy & issue briefs, working papers, databases, videos, training tools, fact sheets
Policy engagement	**Number of training events with journalists, parlia-mentarians, bureaucrats, and civil society organizations**	Do you hold any capacity training events with journalists, parliamentarians, bureaucrats, civil society organizations, and others in an average year? If so, how often do you hold such events?	Yes, at least two capacity building activities with media, one legislative retreat, and several visits to State Houses of Assembly	Yes, 5 times a year	Yes, twice a year	No	Yes, twice a year	Yes, quarterly

The metrics on dissemination and policy engagement activities have a high correlation with the patterns of efficacy observed earlier. CS-SUNN, UNICEF and FHI360 conduct a wide variety of media activities and produce multiple written and visual outputs while also holding regular outreach activities with decision makers and civil society. CS-SUNN in particular targets particular events, such as World Food Day and World Breastfeeding Week, holds ‘Nutrition Tuesdays’ on Twitter, and capacity training with media and legislators. As the CS-SUNN informant noted, ‘Meetings with the legislators had an impact because of their oversight functions…Meeting with the people who also do the budget had an impact because they may otherwise remove the budget [for nutrition] if they are not aware.’^5^ By contrast, KAF and PPMCH lack operational websites, and the former is limited to newspaper op-eds for much of its outreach, which can undermine engagement in low-literacy communities. These patterns are further reinforced in Table 6, which shows that non-advocacy respondents recall attending many more events, engaging in more informal interactions and receiving regular written updates by CS-SUNN and UNICEF, followed by FHI 360.

**Table 6.** Overview of impact of external outreach activities

Organization	Respondent has attended at least one event hosted by organization in the last 12 months *(Number of responses)*	Organizations with which respondent has had informal interactions during the last 12 months *(Number of responses)*	Organizations from which respondent regularly receives updates and outputs by mail or email *(Number of responses)*
FHI360	3	6	6
KAF	–	–	–
NI	–	–	1
UNICEF	10	16	11

Notes: Informal interactions refers to phone calls, unplanned office visits, or casual meetings.

## Discussion

Collectively, the metrics presented in Tables 2 through 5 reinforce that multiple measures are needed to assess when and why some advocacy organizations are perceived as more efficacious than others. No one indicator alone will provide the full story of why some advocates are more visible and impactful. Therefore, a comprehensive and systematic stocktaking of an advocacy organization’s internal capacities, networks, and outreach activities is needed.

At the same time, some metrics were more informative than others to understand variation in efficacy across the six focus organizations, and these were indicated in boldface within the corresponding tables. Specifically, many staff with relevant nutrition skills, a broad geographical reach, sufficient financing devoted to advocacy from extant resources, and an action plan with clear objectives differentiated CS-SUNN and UNICEF and to some extent, FHI360, from the organizational capacity of KAF, NI and PPMCH. Network strength is conveyed by the degree of centrality an organization plays in the nutrition landscape and its integration of powerful government and non-governmental policy champions. For external outreach, using multiple forms of media, ensuring an operational website, producing diverse outputs and holding multiple training events a year increases visibility. When this particular sub-set of metrics are examined together, the reasons why these organizations fall along a spectrum of perceived efficacy by other in-country stakeholders—with CS-SUNN and UNICEF at one end and KAF and PPMCH at the other—becomes more apparent.

Notably, KAF and PPMCH are domestic advocacy organizations but were not identified as top advocacy organizations even by LGA and state-level actors within Kano, where they are based. The application of the same metrics to other domestic organizations, including FOMWAN, TDI and Wazobia, reveals that they fare worse than international ones and are less recognized by other stakeholders. This finding may reflect our narrow definition of advocacy efficacy, which focuses on nutrition policy outcomes and therefore does not capture broader societal achievements; indeed, Kurfu (2018) shows that FOMWAN has been instrumental in enhancing Muslim women’s empowerment in Nigeria. Nevertheless, our analysis vis-à-vis nutrition policy suggests worrisome cyclical effects whereby domestic organizations are overlooked by donors for financial support on nutrition advocacy that potentially would allow them to improve their metrics, therefore causing them to continue to be viewed as less effective. This raises questions about the local ownership of nutrition policy and the balance of power between international and domestic organizations that is increasingly recognized as problematic within public health (Shiffman, 2014; Harris, 2019; Storeng *et al.*, 2019). Simultaneously, CS-SUNN’s ability to achieve a high level of perceived efficacy among policymakers highlights the advantages of its hybrid modality.

Additional validation of the metrics is required in other policy settings where the underlying enabling environment may further streamline the requisites for effective advocacy. In settings with more centralized or more authoritarian decision-making structures, fewer policy champions may be needed or external outreach through multiple media outlets might not be feasible. In other words, as highlighted in Figure 1, the enabling environment will structure how the metrics should be interpreted. By way of example, the relevance of the FMBNP as one of CS-SUNN’s and UNICEF’s perceived policy champions is not obvious without knowing the significance of that ministry in Nigeria’s nutrition policy landscape.

## Conclusion

Neither the importance of advocacy for nutrition policy reforms nor the discussion of some of the advocacy metrics reviewed here, may be surprising. Yet, in practice, there are few attempts to operationalize many of the best principles for nutrition advocacy. Establishing metrics that can be refined, tested over time and applied in different settings can both assist with comparative research on advocacy efforts in a more a systematic way, as well as contribute to organizational learning about gaps that need to be addressed to exert more influence in both the nutrition field and other policy spheres.

## Supplementary Material

czac037_Supp

## Data Availability

The data underlying this article cannot be shared publicly out of respect for the privacy of the individuals who participated in the study and their roles within the advocacy organizations that they represent. The data will be shared on reasonable request to the corresponding author.

## Footnotes

1. KII respondent with independent nutrition consultant, Abuja, November 2019.

2. KII respondent with private sector foundation, Abuja, October 2019.

3. KII with nutrition consultant, Abuja, December 13, 2019.

4. NI used to be named the Micronutrient Initiative.

5. KII with CS-SUNN representative at federal level, Abuja, October 2019.

## Reflexivity statement

The authors include two females and one male and span multiple levels of seniority. While two of the authors specialize in nutrition and health policy in Nigeria and West Africa, the third is a political scientist with expertise in nutrition and food security policy processes in the Global South. All three authors have extensive experience conducting qualitative fieldwork in Nigeria, especially in Kaduna state and the Federal Capital Territory.

## Appendix 1

**Table A1.** List of stakeholders interviewed

Category	Stakeholder
*Federal*	
Advocacy	FHI 360 Civil Society-Scaling Up Nutrition in Nigeria Clinton Health Access Initiative Food and Agricultural Organization Global Alliance for Improved Nutrition Helen Keller International Nutrition International Plan International Save the Children SUN Business Network Technoserve UNICEF
Government	Federal Ministry of Agriculture and Rural Development Federal Competition and Consumer Protection Commission Federal Ministry of Finance, Budget, and National Planning Federal Ministry of Health Standards Organization of Nigeria

**Table A1.** (Continued)

Category	Stakeholder
Donors	Aliko Dangote Foundation European Union Delegation UK Department for International Development World Bank
Research/Consultant	Nutrition consultants
*Kaduna*	
Advocacy	Civil Society-Scaling Up Nutrition in Nigeria Save the Children UNICEF
Government	Primary Health Care Department, Giwa LGA Agriculture and Forestry Sector, Giwa LGA Kaduna State Agricultural Development Agency Kaduna Planning and Budget Commission Kaduna State Emergency Nutrition Action Plan Kaduna State Primary Health Care Development Agency Department of Agriculture, Kachia LGA Primary Health Care Department, Kachia, LGA
Media	Kaduna State Media Corporation
Research/Consultant	Ahmadu Bellow University, Zaria Nutrition consultant
*Kano*	
Advocacy	Kano Nutrition Working Group Transparency and Development Information Initiative Federation of Muslim Women’s Associations in Nigeria Partnership for the Promotion of Maternal and Child Health in Kano State Kola and Funke Care Foundation Wazobia International Women and Children Foundation
Government	State Primary Health Care Management Board Kano Ministry of Planning and Budget Kano Ministry of Health National Orientation Agency Ministry of Agriculture Primary Healthcare Department, Wudil LGA Agriculture and Natural Resources Department, Wudil, LGA Primary Healthcare Department, Bichi LGA Agricultural Department, Bichi LGA
Media	Express Radio Kano Abubakar Rimi Television
Research/Consultants	Bayero University

## Appendix 2

**Table A2.** List of acronyms

Acronym	Name
AMMKaS	Accountability Mechanism for Maternal and Child Health in Kano State
BMGF	Bill and Melinda Gates Foundation
CHAI	Clinton Health Access Initiative
CISLAC	Civil Society Legislative Advocacy Center
CS-SUNN	Civil Society-Scaling Up Nutrition in Nigeria
FAO	Food and Agricultural Organization
FHI360	Family Health International 360
FMARD	Federal Ministry of Agriculture and Rural Development
FMBNP	Federal Ministry of Budget and National Planning
FMOH	Federal Ministry of Health
FOWMAN	Federation of Muslim Women’s Associations in Nigeria
GAIN	Global Alliance for Improved Nutrition
HISP	Health Information Systems Nigeria
HKI	Helen Keller International
IYCF	Infant and young child feeding
ISMPH	International Society of Media in Public Health
JAM	Journalists Against Malnutrition
KAF	Kola and Funke Care Foundation
KNWG	Kano Nutrition Working Group
LHI	Life Helpers International
MBNP	Ministry of Budget and National Planning (State Level)
MNCH2	Maternal, Newborn, and Child Health Program
NAFDAC	National Agency for Food and Drug Administration
NI	Nutrition International
NOA	National Orientation Agency
NSN	Nutrition Society of Nigeria
NSTOP	National Stop Transmission of Polio
PI	Plan International
PPMCH	Partnership for the Promotion of Maternal and Child Health in Kano State
SFH	Society for Family Health
SOML	Saving One Million Lives
SON	Standards Organization of Nigeria
SuNMaP	Support to the National Malaria Program
TDII	Transparency and Development Information Initiative
UNFPA	United Nations Population Fund
UNICEF	United Nations International Children’s Fund
WDC	Ward Development Committee
WF	Wellbeing Foundation
WFP	World Food Program
WISH	Women Integrated Services for Health
