# Supplementary material for: Advocacy organizations and nutrition policy in Nigeria: identifying metrics for enhanced efficacy
Source: Health Policy Plan. 2022 Apr 28;37(8):963–78. doi: 10.1093/heapol/czac037 (PMC9469884; doi:10.1093/heapol/czac037)
Supplement: czac037_Supp [file czac037_supp.zip › Appendix 1.docx]

**Appendix 1: List of Stakeholders Interviewed**

| **Category** | **Stakeholder** |
| --- | --- |
| ***Federal*** |  |
| Advocacy | - FHI 360 - Civil Society-Scaling Up Nutrition in Nigeria - Clinton Health Access Initiative - Food and Agricultural Organization - Global Alliance for Improved Nutrition - Helen Keller International - Nutrition International - Plan International - Save the Children - SUN Business Network - Technoserve - UNICEF |
| Government | - Federal Ministry of Agriculture and Rural Development - Federal Competition and Consumer Protection Commission - Federal Ministry of Finance, Budget, and National Planning - Federal Ministry of Health - Standards Organization of Nigeria |
| Donors | - Aliko Dangote Foundation - European Union Delegation - UK Department for International Development - World Bank |
| Research/Consultant | - Nutrition consultants |
| ***Kaduna*** |  |
| Advocacy | - Civil Society-Scaling Up Nutrition in Nigeria - Save the Children - UNICEF |
| Government | - Primary Health Care Department, Giwa LGA - Agriculture and Forestry Sector, Giwa LGA - Kaduna State Agricultural Development Agency - Kaduna Planning and Budget Commission - Kaduna State Emergency Nutrition Action Plan - Kaduna State Primary Health Care Development Agency - Department of Agriculture, Kachia LGA - Primary Health Care Department, Kachia, LGA |
| Media | - Kaduna State Media Corporation |
| Research/  Consultant | - Ahmadu Bellow University, Zaria - Nutrition consultant |
| ***Kano*** |  |
| Advocacy | - Kano Nutrition Working Group - Transparency and Development Information Initiative - Federation of Muslim Women’s Associations in Nigeria - Partnership for the Promotion of Maternal and Child Health in Kano State - Kola and Funke Care Foundation - Wazobia International Women and Children Foundation |
| Government | - State Primary Health Care Management Board - Kano Ministry of Planning and Budget - Kano Ministry of Health - National Orientation Agency - Ministry of Agriculture - Primary Healthcare Department, Wudil LGA - Agriculture and Natural Resources Department, Wudil, LGA - Primary Healthcare Department, Bichi LGA - Agricultural Department, Bichi LGA |
| Media | - Express Radio Kano - Abubakar Rimi Television |
| Research/Consultants | - Bayero University |
