# Supplementary material for: Advocacy organizations and nutrition policy in Nigeria: identifying metrics for enhanced efficacy
Source: Health Policy Plan. 2022 Apr 28;37(8):963–78. doi: 10.1093/heapol/czac037 (PMC9469884; doi:10.1093/heapol/czac037)
Supplement: czac037_Supp [file czac037_supp.zip › Appendix 2.docx]

**Appendix 2: List of acronyms**

| **Acronym** | **Name** |
| --- | --- |
| AMMKaS | Accountability Mechanism for Maternal and Child Health in Kano State |
| BMGF | Bill and Melinda Gates Foundation |
| CHAI | Clinton Health Access Initiative |
| CISLAC | Civil Society Legislative Advocacy Center |
| CS-SUNN | Civil Society-Scaling Up Nutrition in Nigeria |
| FAO | Food and Agricultural Organization |
| FHI360 | Family Health International 360 |
| FMARD | Federal Ministry of Agriculture and Rural Development |
| FMBNP | Federal Ministry of Budget and National Planning |
| FMOH | Federal Ministry of Health |
| FOWMAN | Federation of Muslim Women’s Associations in Nigeria |
| GAIN | Global Alliance for Improved Nutrition |
| HISP | Health Information Systems Nigeria |
| HKI | Helen Keller International |
| IYCF | Infant and young child feeding |
| ISMPH | International Society of Media in Public Health |
| JAM | Journalists Against Malnutrition |
| KAF | Kola and Funke Care Foundation |
| KNWG | Kano Nutrition Working Group |
| LHI | Life Helpers International |
| MBNP | Ministry of Budget and National Planning (State Level) |
| MNCH2 | Maternal, Newborn, and Child Health Program |
| NAFDAC | National Agency for Food and Drug Administration |
| NI | Nutrition International |
| NOA | National Orientation Agency |
| NSN | Nutrition Society of Nigeria |
| NSTOP | National Stop Transmission of Polio |
| PI | Plan International |
| PPMCH | Partnership for the Promotion of Maternal and Child Health in Kano State |
| SFH | Society for Family Health |
| SOML | Saving One Million Lives |
| SON | Standards Organization of Nigeria |
| SuNMaP | Support to the National Malaria Program |
| TDII | Transparency and Development Information Initiative |
| UNFPA | United Nations Population Fund |
| UNICEF | United Nations International Children’s Fund |
| WDC | Ward Development Committee |
| WF | Wellbeing Foundation |
| WFP | World Food Program |
| WISH | Women Integrated Services for Health |
